# The impact of histological subtype on postoperative recurrence pattern and timing in locally advanced esophagogastric junction cancer

**DOI:** 10.1007/s12672-024-01353-x

**Published:** 2024-09-19

**Authors:** Shinsuke Maeda, Masaho Ota, Shunichi Ito, Kei Hosoda

**Affiliations:** 1 Department of Surgery, Institute of Gastroenterology, Tokyo Women’s Medical University, 8-1, Kawadacho, Shinjuku-ku, Tokyo 162-8666 Japan; 2 Division of Gastroenterological Surgery, Tokyo Women’s Medical University Yachiyo Medical Center, Chiba, Japan

**Keywords:** Esophagogastric junction, Histological subtype, Recurrence pattern, Recurrence timing

## Abstract

**Purpose:**

The differences in tumor behavior between adenocarcinoma (AC) and squamous cell carcinoma (SCC) of the esophagogastric junction (EGJ) have yet to be well investigated. The purpose of this study was to gain insights that can contribute to tailored treatments and follow-up strategies by analyzing the correlation between histological subtypes and oncological outcomes.

**Methods:**

A retrospective analysis was used to determine the characteristics of the histological subtypes of EGJ cancer by comparing the appearance of postoperative recurrence. A total of 102 consecutive patients with pathological stage IIA to IVA EGJ cancer, who underwent R0 surgery in our department from 2004 to 2020, were enrolled. The recurrence pattern, timing, survival, and potential prognostic factors were compared.

**Results:**

After a median follow-up time of 70.1 months, the AC group demonstrated comparable lymph node failure-free survival (P = 0.291) and significantly worse non-lymphogenous recurrence-free survival (P = 0.035) than did the SCC group. A significantly longer period from surgery to recurrence was also observed in the AC group (P = 0.029). Multivariate analysis indicated that histological subtype (P = 0.015, 95% CI 1.24–7.28) was significantly correlated with the incidence of non-lymphogenous recurrence.

**Conclusions:**

The pattern and timing of postoperative recurrence were significantly different between the histological subtypes of EGJ cancer. Compared with EGJ SCC, EGJ AC may have a greater tendency toward non-lymphogenous progression and a greater propensity for longer surgery-to-recurrence periods.

## Introduction

In 2020, carcinoma of the esophagus was recorded as the seventh most frequent type of cancer (604,000 new cases) and the sixth most common cause of cancer-related mortality (544,000 deaths) worldwide [1]. Although squamous cell carcinoma (SCC) is currently the main subtype of esophageal cancer, the incidence rate of esophageal and esophagogastric junction (EGJ) adenocarcinoma (AC) is currently on the rise in high-income countries, including Japan [2–4]. Furthermore, currently available treatment strategies for this type of tumor are highly controversial. The European Society for Medical Oncology (ESMO) clinical practice guidelines for esophageal cancer recommend both perioperative chemotherapy and preoperative chemoradiotherapy (CRT) for resectable locally advanced AC, but controversy exists regarding which of the two treatments is the better choice [5]. For resectable locally advanced SCC, preoperative CRT is the only recommended treatment option for perioperative therapy in Western countries. In Japan, however, the NExT study did not demonstrate the superiority of preoperative CRT compared to preoperative doublet CF (cisplatin plus 5-FU) therapy [6]. As a result, preoperative triplet DCF (docetaxel, cisplatin, plus 5-FU) therapy has emerged as the primary recommendation.

Esophageal SCC and AC have often been treated as single entities in clinical studies, and few studies have investigated the differences in oncological behavior between these histological subtypes. Understanding the differences in oncological behavior may contribute to the development of a more personalized therapeutic strategy. Thus, in this study, a retrospective investigation was conducted on the appearance of recurrence after surgery for EGJ cancer in relation to histological subtypes.

## Materials and methods

### Patient selection

A retrospective analysis was conducted on 150 consecutive patients with EGJ carcinoma who underwent surgical resection at Tokyo Women’s Medical University Hospital (Tokyo, Japan) between January 2004 and October 2020. EGJ carcinoma was defined according to Nishi’s classification [7]. Tumors with their epicenter located between 2 cm proximal and distal from the anatomical EGJ, irrespective of histology, were classified as EGJ carcinomas. The anatomical EGJ was identified from the resected specimen and was defined as the distal end of the palisade vessels or deep esophageal glands and ducts. When the EGJ could not be detected microscopically due to tumor invasion, the level of macroscopic caliber change was used to define the EGJ.

All patients underwent pretreatment evaluation, which included esophagogastroduodenoscopy (EGD) with biopsy, computed tomography (CT) from the chest to the pelvis, and/or positron emission tomography (PET). Staging was classified according to the 8th edition of the TNM staging system of the Union for International Cancer Control. The inclusion criteria were as follows: (1) histologically diagnosed squamous cell carcinoma (including basaloid squamous cell carcinoma) or adenocarcinoma, except for adenosquamous carcinoma; (2) no residual tumor under microscope (R0); (3) no concomitant or prior malignancy within 5 years; (4) no history of preoperative endoscopic resection of the primary lesion; (5) no M1 disease at the time of initial diagnosis; (6) pathological stage IIA to IVA (T2-4 or N( +), except for M1); and (7) follow-up with periodic CT and EGD after surgery. This study was approved by the Ethics Review Board of Tokyo Women’s Medical University (Approval number: 4582).

### Surgery and perioperative treatment

The choice of surgical procedure was determined by the length of esophageal involvement, the general condition of the patients, and the experience of the surgeons. For ACs with esophageal involvement greater than 3 cm, transthoracic approach was commonly selected. For ACs with esophageal involvement less than 3 cm, transabdominal lower esophagectomy with proximal or total gastrectomy was prevalently performed. For SCC, transthoracic surgery was performed in principle. For abdominal lymphadenectomy, the paracardial; along the left gastric artery; the lesser curvature lymph nodes (LNs) along the branches of the left gastric artery; the esophageal hiatal; and clinically positive LNs were routinely resected. Mediastinal lymphadenectomy included middle and lower thoracic periesophageal and subcarinal LNs when a transthoracic approach was used; the lower mediastinal nodes up to the level of the proximal margin were included with a transabdominal approach. The extent of further lymphadenectomy (e.g., recurrent nerve LNs) depended on the preoperative findings. Digestive tract reconstruction involved the stomach or jejunum. The Clavien-Dindo (CD) classification system was used to assess postoperative complications [8]. Patients with Grade ≥ 2 complications were classified as having complications.

For patients with an estimated high risk of postoperative relapse (i.e., T-category > 3 and/or positive N status) and with tolerable organ functions, platinum- or taxane-based neoadjuvant chemotherapy with or without concurrent 40 Gy of radiotherapy and/or adjuvant chemotherapy was administered. More than two-thirds of the regimens for the respective group of patients were adjuvant oral pyrimidine fluoride with standard period of one year for the AC patients and neoadjuvant or adjuvant platinum plus pyrimidine fluoride with standard courses of two for the SCC patients.

### Postoperative surveillance

After surgery, the patients were followed up every three months for the first two years, semiannually for the next three years, and annually thereafter. During follow-up, blood tests and a CT scan from the chest to the pelvis were performed at every scheduled interval. EGD was conducted annually. When cancer recurrence was suspected on CT images or laboratory data, PET was additionally performed.

### Pattern of recurrence

Recurrence was diagnosed with histological, cytological, or definite radiological evidence. Only the first episode of recurrence was used for analysis. For patients with concurrent multifocal recurrences, each categorized pattern was counted. The local area was defined to include the tumor bed, anastomosis, remnant esophagus, and remnant stomach. The area of group 1 and 2 LNs of the Ae (zone of the EGJ) regional tumor according to the 11th edition of the Japanese Classification of Esophageal Cancer was defined as the regional area [9].

The patterns of recurrence were first categorized into local recurrence, regional LN metastasis, distant LN metastasis, distant organ metastasis, and peritoneal or pleural dissemination. Locoregional recurrence (LRR) and systemic metastasis (SM) were subsequently classified. Similarly, recurrences other than LN failure were classified as non-lymphogenous recurrences (NLRs). The data were last updated in February 2023.

### Statistical analysis

Continuous variables were compared using Welch’s t-test. Categorical variables were compared using the chi-square test. Age, primary tumor length, and number of examined LNs were grouped by the median value as a cutoff. Survival times were defined from the date of surgery. The timing of recurrence was calculated from the day of surgery to the day of the first event. The Kaplan–Meier method was used to analyze survival times, and the log-rank test was used to examine intergroup differences. A univariate Cox proportional hazards regression model was used to analyze prognostic factors, and a Fine-Gray proportional hazards regression model was used in the multivariate analysis. Covariates with P < 0.1 in the univariate analysis (histological subtype, pathological N-category, pathological TNM-stage, lymphatic microinvasion, and differentiation) were entered into the multivariate analysis and the backward stepwise method was used to optimize the model. Statistical analyses were conducted using R software version 4.1.2 (The R Foundation for Statistical Computing, Vienna, Austria). P < 0.05 was considered to indicate statistical significance.

## Results

### Patient characteristics

**Fig. 1:**
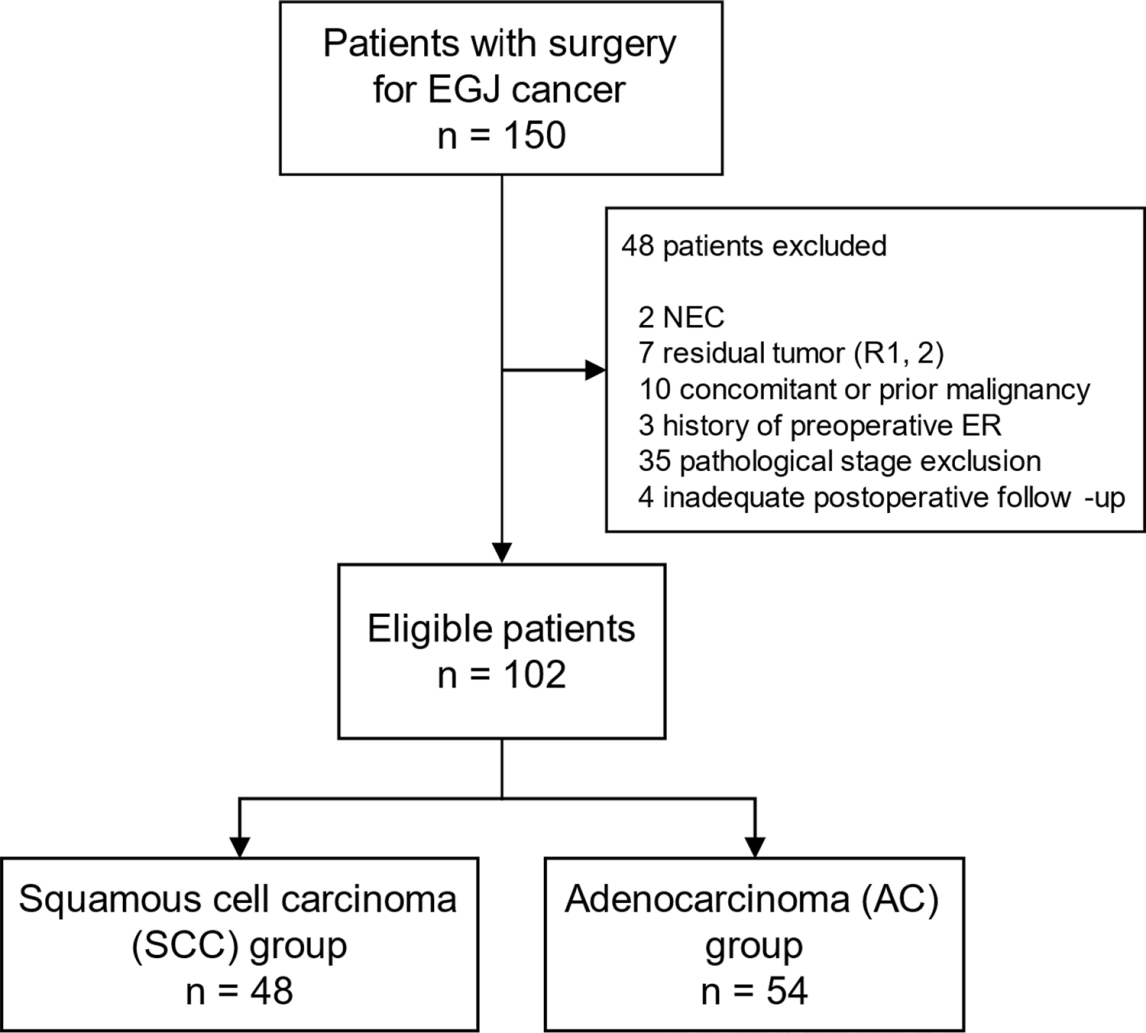
Flow diagram of patient selection. *EGJ* esophagogastric junction, *NEC* neuroendocrine carcinoma, *ER* endoscopic resection

A total of 102 eligible patients with EGJ cancer were analyzed: 48 with SCC and 54 with AC (Fig. 1). The median age of the whole group was 67 years (range, 42–85 years), and the median longitudinal length of the primary tumor in the resected specimen was 53 mm (range, 10–110 mm). A summary of the comparisons between the histology groups is provided in Table 1. An analysis of the patient characteristics revealed no significant intergroup differences in terms of age; tumor diameter or location; number of metastatic LNs; pathological T-category, N-category, or TNM-stage; lymphatic and venous microinvasions; differentiation of tumor cells; or incidence of postoperative complications. Compared to patients with SCC, patients with AC were more frequently male (88.8% vs 64.6%; P = 0.007) and underwent transabdominal surgery more often (38.9% vs 6.3%; P < 0.001). Accordingly, a greater number of LNs were examined in the SCC group (mean number = 21.7 vs 27.9; P = 0.019). Combined distal pancreatectomy and splenectomy due to tumor invasion was performed in 2 pathological stage IVA patients with SCC. A total of 68 patients (66.7%) were administered perioperative treatment. Adjuvant treatment was more frequently administered for ACs (57.4% vs 35.4%; P = 0.043), although the frequency of neoadjuvant treatment was not significantly different (16.7% vs 31.3%; P = 0.134). The overall frequency of perioperative treatment was comparable (68.5% vs 64.6%; P = 0.833).

**Table 1:** Background characteristics and pathological findings of the patients

Characteristic	All patients (n = 102)	SCC (n = 48)	AC (n = 54)	p Value
	n (%)	n (%)	n (%)	
Age, years				0.094
Median	67	69.5	67	
Range	42–85	48–85	42–84	
Gender				**0.007**
Male	79 (77.5)	31 (63.5)	48 (88.9)	
Female	23 (22.5)	17 (35.4)	6 (11.1)	
Tumor diameter, mm				0.549
Median	53	52.5	53	
Range	10–110	10–105	16–110	
Tumor location				0.269
Across the EGJ	94 (92.2)	43 (89.6)	51 (94.4)	
Esophagus only	7 (6.9)	5 (10.4)	2 (3.7)	
Stomach only	1 (1.0)	0 (0.0)	1 (1.9)	
Surgical procedure				** < 0.001**
Transthoracic	78 (76.5)	45 (93.8)	33 (61.1)	
Transabdominal	24 (23.5)	3 (6.2)	21 (38.9)	
Number of examined LNs (mean ± SD)	24.6 ± 13.1	27.9 ± 14.1	21.7 ± 11.5	**0.019**
Number of metastatic LNs (mean ± SD)	3.10 ± 3.59	2.60 ± 2.83	3.54 ± 4.12	0.182
Postoperative complication^a^				1.00
Yes	21 (20.6)	10 (20.8)	11 (20.4)	
No	81 (79.4)	38 (79.2)	43 (79.6)	
Perioperative treatment				0.833
Yes	68 (66.7)	31 (64.6)	37 (68.5)	
No	34 (33.3)	17 (35.4)	17 (31.5)	
Neoadjuvant treatment				0.134
Yes	24 (23.5)	15 (31.3)	9 (16.7)	
No	78 (76.5)	33 (68.8)	45 (83.3)	
Adjuvant treatment				**0.043**
Yes	48 (47.1)	17 (35.4)	31 (57.4)	
No	54 (52.9)	31 (64.6)	23 (42.6)	
Pathological T-category^b^				0.378
1	8 (78.4)	3 (6.3)	5 (9.3)	
2	11 (10.8)	4 (3.9)	7 (13.0)	
3	81 (79.4)	39 (81.3)	42 (77.8)	
4	2 (2.0)	2 (4.2)	0 (0.0)	
Pathological N-category^b^				0.164
0	30 (29.4)	14 (29.2)	16 (29.6)	
1	32 (31.4)	15 (14.7)	17 (35.4)	
2	22 (21.6)	14 (29.2)	8 (14.8)	
3	18 (17.6)	5 (4.9)	13 (24.1)	
Pathological TNM-stage^b^				0.150
IIA	7 (6.9)	4 (8.3)	3 (5.6)	
IIB	29 (28.4)	12 (25.0)	17 (31.5)	
IIIA	6 (5.9)	1 (2.1)	5 (9.3)	
IIIB	40 (39.2)	24 (50.0)	16 (29.6)	
IVA	20 (19.6)	7 (14.6)	13 (24.1)	
Lymphatic microinvasion				1.00
None or slight	47 (46.1)	22 (45.8)	25 (46.3)	
Moderate or severe	55 (53.9)	26 (54.2)	29 (53.7)	
Venous microinvasion				0.948
None or slight	73 (71.6)	35 (72.9)	38 (70.4)	
Moderate or severe	29 (28.4)	13 (27.1)	16 (29.6)	
Differentiation of tumor cells				0.530
Well or moderate	68 (66.7)	34 (70.8)	34 (63.0)	
Poor	34 (33.3)	14 (29.2)	20 (37.0)	

Bold font indicates significance

*SCC* squamous cell carcinoma, *AC* adenocarcinoma, *EGJ* esophagogastric junction, *LN* lymph node, *CD* Clavien-Dindo

^a^Grade ≥ 2 according to the Clavien-Dindo (CD) classification system

^b^The 8th edition of the TNM staging system of the Union for International Cancer Control (UICC)

### Recurrence pattern

**Fig. 2:**
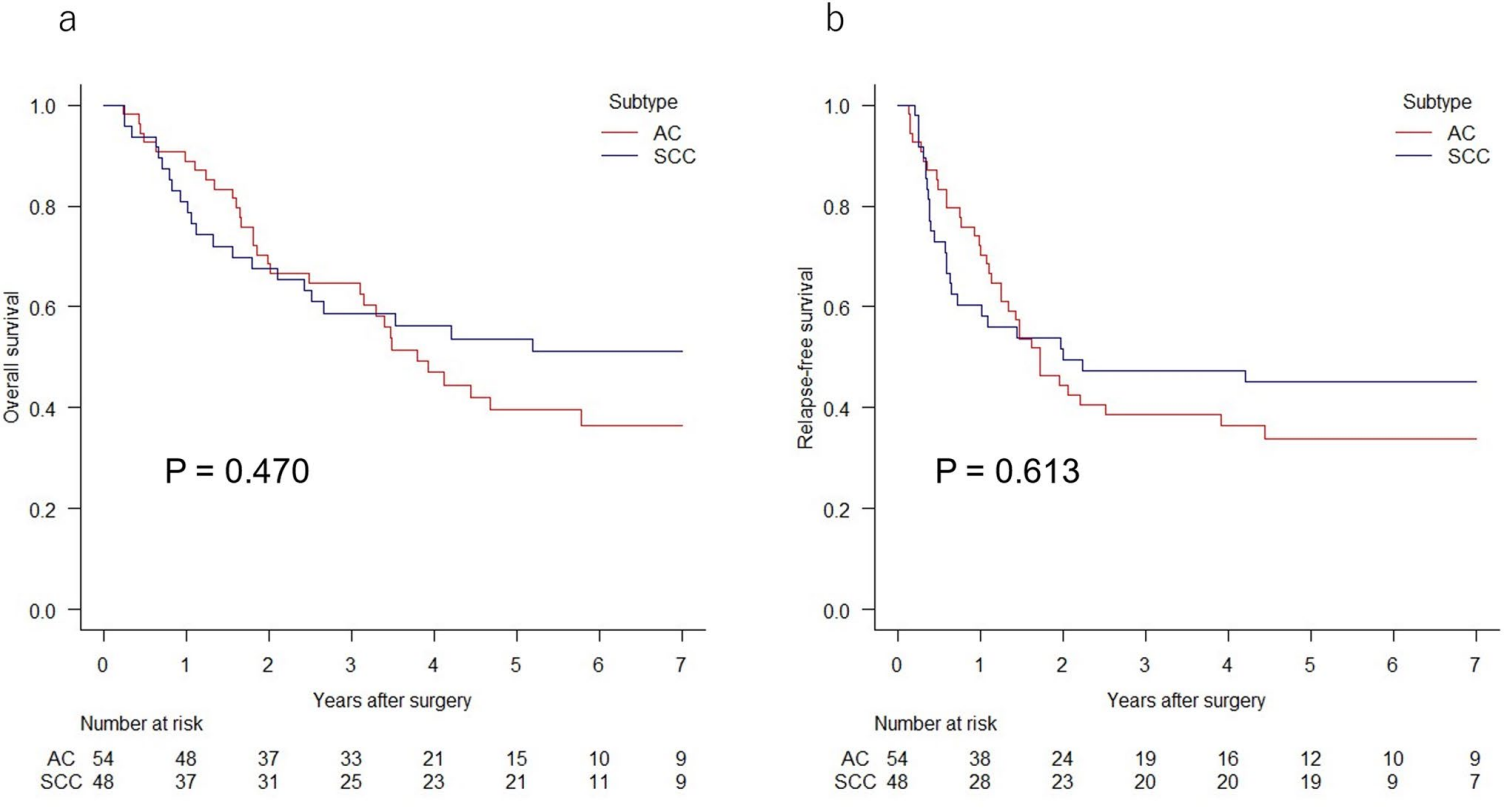
Kaplan–Meier curves for overall survival **a** and relapse-free survival **b** for patients with esophagogastric junction adenocarcinoma (AC) or squamous cell carcinoma (SCC)

**Fig. 3:**
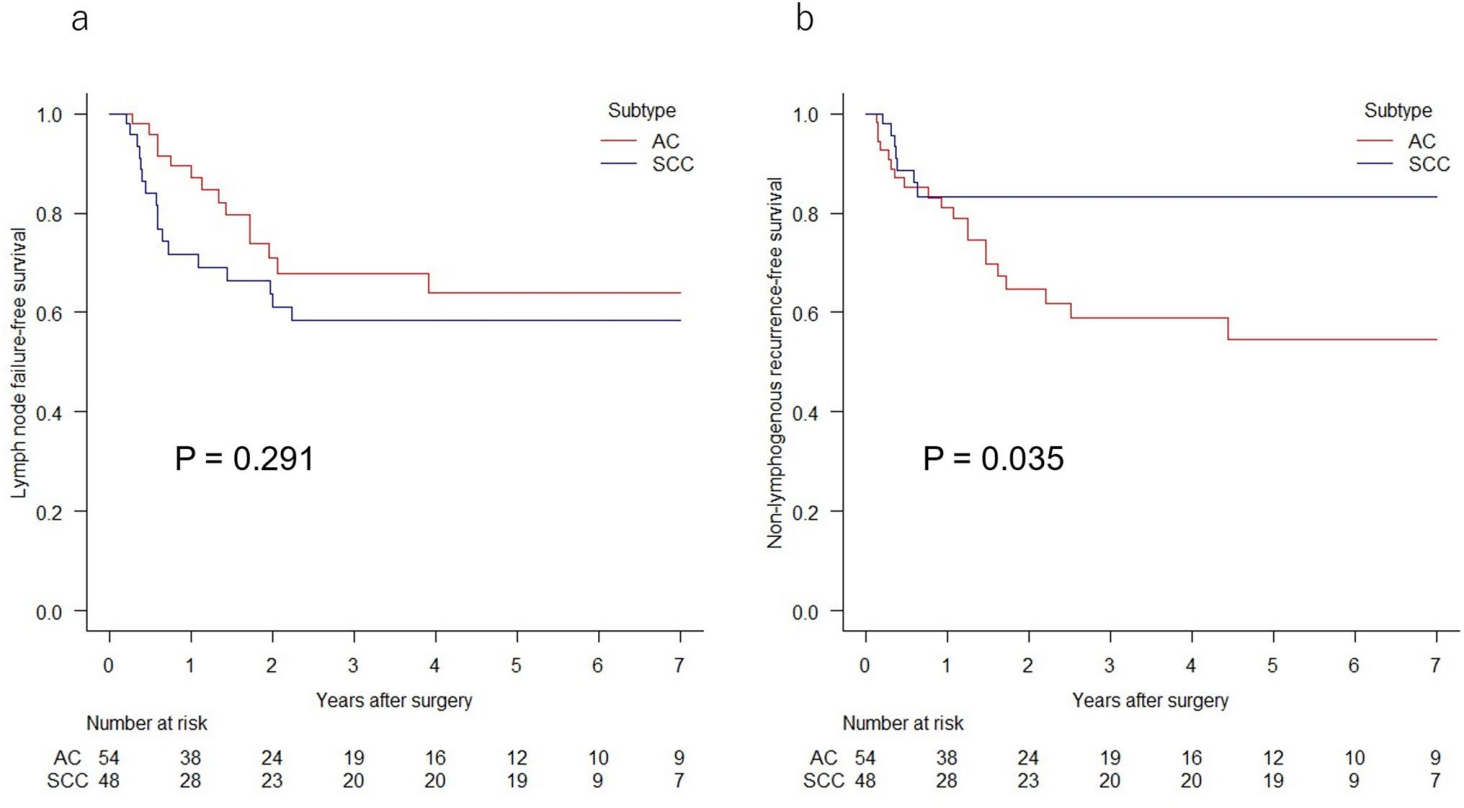
Kaplan–Meier curves for lymph node failure-free survival **a** and non-lymphogenous recurrence-free survival **b** in patients with esophagogastric junction adenocarcinoma (AC) or squamous cell carcinoma (SCC)

After a median follow-up time of 70.1 months (interquartile range [IQR], 67.1–95.0 months) for SCC and 70.9 months (IQR, 43.9–98.7 months) for AC, 21 patients (43.8%) with SCC and 33 patients (61.1%) with AC experienced recurrence. The 5-year overall survival (OS) and relapse-free survival (RFS) rates for patients with SCC vs. AC were 53.7 vs. 39.6% (P = 0.470; Fig. 2a) and 45.0 vs. 33.8% (P = 0.613; Fig. 2b), respectively. Ratio of recurrence in the subgroups of pathological stage II, III, and IVA patients were 12.5, 52.0, and 85.7% in the SCC group, and were 40.0, 71.4, and 76.9% in the AC group, respectively. A total of 17 patients (35.4%, 7 above and 11 below the diaphragm) experienced LN failure, and 7 (14.6%) had NLRs among patients with SCC. In the AC group, 14 patients (25.9%, 6 above and 8 below the diaphragm) experienced LN failure, and 20 (37.0%) had NLR (Table 2). The AC group demonstrated comparable LN failure-free survival (P = 0.291; Fig. 3a) and significantly worse NLR-free survival (P = 0.035; Fig. 3b) than did the SCC group.

**Table 2:** Recurrence patterns

	SCC (n = 48)	AC (n = 54)
No. of patients with any relapse: n (%)	21 (43.8)	33 (61.1)
Lymphogenous: n (%)	17 (35.4)	14 (25.9)
Regional LN	7	3
(Above/below the diaphragm)	(2/5)	(1/2)
Distant LN	14	12
(above/below the diaphragm)	(7/7)	(6/6)
Non-lymphogenous: n (%)	**7 (14.6)**^**a**^	**20 (37.0)**^**a**^
Local	2	6
Anastomosis	2	0
Tumor bed	0	4
Remnant esophagus	0	2
Systemic	5	15
Liver	2	4
Lung	1	2
Bone	0	1
Adrenal	1	0
Heart	0	1
Pleura	1	4
Peritoneum	0	4

Bold font indicates significance

*SCC* squamous cell carcinoma, *AC* adenocarcinoma, *LN* lymph node

^a^p < 0.05

Regarding the areas of relapse, 9 (18.8%) LRRs and 17 (35.4%) SMs occurred in the SCC group, and 9 (16.7%) LRRs and 27 (50.0%) SMs were observed in the AC group. Both LRR-free survival and SM-free survival were comparable between the histological subtypes (P = 0.696 and 0.339, respectively). The specific sites of recurrence are listed in Table 2. The liver was the most common site of distant metastasis in both groups. Both pleural and peritoneal carcinomatosis were frequent in the AC group, and the rate of dissemination was greater in the AC group, although the difference was not significant (14.8% vs 2.1%; P = 0.063).

### Recurrence timing

For patients who experienced recurrence, the median time to the first recurrence was 13.9 months (IQR, 5.9–20.9 months) after surgery in the AC group and 7.0 months (IQR, 4.5–8.7 months) in the SCC group. A significantly longer period from surgery to recurrence was observed in the AC group (P = 0.029). Overall, 76.2% of the first recurrences in patients with SCC versus 45.5% in those with AC occurred within one year of follow-up. The surgery-to-NLR periods were significantly longer in the AC group (median, 12.2 vs 4.5 months; IQR, 3.8–18.4 vs 4.2–6.0 months; P = 0.007), whereas the surgery-to-LN failure periods were comparable between the two groups (median, 15.1 vs 7.2 months; IQR, 7.7–20.9 vs 4.7–13.3 months; P = 0.095). Neither the surgical approach nor the administration of perioperative treatment was significantly correlated with recurrence timing. The timing and frequency of recurrence are listed in Table 3.

**Table 3:** Timing and frequency of recurrence

	Total	Incidence	≤12.0 months	12.1–24.0 months	24.1–36.0 months	36.1–48.0 months	48.1–60.0 months	≥60.1 months
	n	n (%)	n (%)	n (%)	n (%)	n (%)	n (%)	n (%)
Total recurrences								
SCC	48	21 (43.8)	16 (76.2)	4 (19.0)	1 (4.8)	0 (0.0)	0 (0.0)	0 (0.0)
AC	54	33 (61.1)	15 (45.5)	13 (39.4)	3 (9.1)	1 (3.0)	1 (3.0)	0 (0.0)
Lymph node recurrences								
SCC	48	17 (35.4)	12 (70.6)	4 (23.5)	1 (5.9)	0 (0.0)	0 (0.0)	0 (0.0)
AC	54	14 (25.9)	6 (42.9)	6 (42.9)	1 (7.1)	0 (0.0)	1 (7.1)	0 (0.0)
Non-lymphogenous recurrences								
SCC	48	**7 (14.6)**^**a**^	7 (100)	0 (0.0)	0 (0.0)	0 (0.0)	0 (0.0)	0 (0.0)
AC	54	**20 (37.0)**^**a**^	10 (50.0)	7 (35.0)	2 (10.0)	0 (0.0)	1 (5.0)	0 (0.0)

Bold font indicates significance

*SCC* squamous cell carcinoma, *AC* adenocarcinoma

^a^p < 0.05

### Prognostic factors for recurrence

Univariate and multivariate analyses of prognostic factors for developing NLR were performed (Table 4). Multivariate analysis revealed that histological subtype (P = 0.015, 95% CI  1.24–7.28), pathological TNM-stage (P = 0.002, 95% CI 1.59–7.17), and differentiation (P = 0.029, 95% CI 1.09–4.89) were independent predictive factors of NLR.

**Table 4:** Univariate and multivariate analyses for non-lymphogenous recurrence

Variable	n	Incidence n (%)	Univariate		Multivariate	
			Hazard ratio (95% CI)	p Value	Hazard ratio (95% CI)	p Value
Age < 68/ ≥ 68	52/50	19 (36.5)/12 (24.0)	0.63 (0.29–1.37)	0.244		
Gender male/female	79/23	23 (29.1)/4 (17.4)	0.62 (0.22–1.81)	0.385		
Histological subtype SCC/AC	48/54	7 (14.6)/20 (37.0)	2.45 (1.04–5.81)	**0.041**	3.00 (1.24–7.28)	**0.015**
Tumor length ≤ 5.0/ > 5.0 cm	47/55	11 (23.4)/16 (29.1)	1.43 (0.67–3.09)	0.358		
Surgical approach transhiatal/transthoracic	24/78	9 (37.5)/18 (23.1)	0.63 (0.28–1.40)	0.257		
Number of examined LNs < 24/ ≥ 24	52/50	14 (26.9)/13 (26.0)	0.94 (0.44–2.01)	0.883		
Postoperative complication^a^ − / +	81/21	21 (25.9)/6 (28.5)	1.15 (0.46–2.85)	0.765		
Neoadjuvant treatment − / +	78/24	19 (24.4)/8 (33.3)	1.63 (0.71–3.72)	0.249		
Adjuvant treatment − / +	54/48	12 (22.2)/15 (31.3)	1.44 (0.67–3.08)	0.346		
Pathological T-category^b^ T1-2 / T3-4	19/83	3 (15.8)/24 (28.9)	2.41 (0.72–8.01)	0.152		
Pathological N-category^b^ N0-1/N2-3	62/40	13 (21.0)/14 (35.0)	2.73 (1.26–5.89)	**0.011**		
Pathological TNM-stage^b^ IIA-IIIA/IIIB-IVA	42/60	7 (16.7)/20 (33.3)	3.12 (1.31–7.44)	**0.010**	3.37 (1.59–7.17)	**0.002**
Lymphatic microinvasion none or slight/mod or more	47/55	7 (14.9)/20 (36.4)	3.48 (1.46–8.26)	**0.005**		
Venous microinvasion none or slight/mod or more	73/29	17 (23.3)/10 (34.5)	1.85 (0.84–4.07)	0.125		
Differentiation well or moderate/other	68/34	13 (19.1)/14 (41.2)	2.52 (1.19–5.38)	**0.016**	2.31 (1.09–4.89)	**0.029**

Bold font indicates significance

*SCC* squamous cell carcinoma, *AC* adenocarcinoma, *CD* Clavien-Dindo

^a^Grade ≥ 2 according to the Clavien-Dindo (CD) classification system

^b^The 8th edition of the TNM staging system of the Union for International Cancer Control (UICC)

LN failure, LRR, and SM did not indicate correlation between histological subtypes. Both neoadjuvant and adjuvant treatments adversely and similarly influenced on incidence of both LN failure (hazard ratio, 1.95 and 2.15) and NLR (hazard ratio, 1.63 and 1.44) on univariate analyses.

## Discussion

Esophageal and EGJ ACs have shown the highest rates of increase in incidence of any cancer over the past four decades, especially in high-income countries [2]. On the other hand, esophageal SCC accounts for up to 90% of cases of esophageal cancer worldwide; however, it is decreasing in Western countries [5, 10]. These two distinct tumors tend to be considered to share similar characteristics and their differences have rarely been investigated. However, in addition to the previously observed greater male predominance of esophageal AC than esophageal SCC, other differences may also exist [11].

Regarding differences in post-therapeutic outcomes, Siewert et al. reported a worse long-term survival among esophageal SCC patients than among AC patients who underwent surgery alone [12]. Thereafter, the CROSS trial demonstrated similar LRR rates between the two histological groups of esophageal or EGJ cancer patients who received neoadjuvant CRT followed by surgery, whereas a higher risk of LRR for SCC was observed in patients who only underwent surgery [13]. Regarding systemic failure, conflicting outcomes regarding its likeliness in relation to histology, caused by varied protocols of trimodality therapy, have been reported [14, 15]. Concerning treatment modalities other than surgery-based treatment, Xi et al. retrospectively compared esophageal cancer patients treated by definitive CRT and reported significantly more favorable hematogenous failure-free survival in SCC patients and significantly greater rates of distant failure in AC patients [16]. However, the locational distributions of the two histological subtypes can vary, and previous studies present a bias in terms of tumor location between the two histological subtypes.

This study is the first to compare postoperative recurrence patterns and timing between AC and SCC patients in an EGJ-limited cohort and to identify differences. We used Nishi's classification in this study since it is applicable for both SCC and AC of the EGJ, unlike Siewert's classification for AC of the EGJ which is commonly used in Western countries. Esophageal SCC occasionally occurs at the zone of the EGJ in Japan, where more than eighty percent of esophageal cancer cases were SCC at the time of 2014 [17]. In this situation, we could select close numbers of eligible EGJ AC and SCC patients, and a significantly greater incidence of postoperative non-lymphogenous failure and a significantly longer period from surgery to recurrence were observed in the AC group than in the SCC group. In contrast, a relatively greater rate of recurrence at the LN was observed in the SCC group, even though the SCC group had a greater number of harvested LNs and a smaller number of metastatic LNs. Traditionally, the spread of a cancer has been stratified as locoregional or systemic in the field of gastroenterology. This concept is suitable for treatments such as surgery or radiotherapy, which are performed to control the locoregional field. However, when a cancer often recurs despite locoregional curative treatment, another viewpoint, such as the route of cancer progression, may be helpful for developing an enhanced therapeutic strategy. Studies involving larger cohorts that focused on individual histological subtypes have shown that the primary cause of postoperative failure in esophageal SCC is often LN metastasis [18]. Conversely, LN metastasis was a less common factor contributing to postoperative failure in EGJ AC [19]. The findings of the present study agree with those of previous studies, suggesting that assessing tumor spreading appearance based on a route-specific viewpoint of lymphogenous or non-lymphogenous, rather than focusing solely on locoregional or systemic aspects may provide more informative insights.

Concerning recurrence timing, the median time-to-recurrence after surgery for esophageal and EGJ AC has been reported to be within a range of 11 to 26 months [20–22]. In esophageal SCC, Kurogochi et al. reported that more than 70% of postoperative recurrences occurred within 1 year [23]. To the best of our knowledge, there have been no direct comparisons of the timing of postoperative recurrence between histological subtypes of esophageal or EGJ cancer. Yun et al. directly compared the timing of recurrence after surgery for non-small cell lung cancer and reported various recurrence hazard peaks depending on tumor histology: 9 months in SCC and 15 months in AC [24]. These findings closely resemble our results, suggesting that longer periods until recurrence in AC than in SCC may be common tumoral characteristics across primary organs, considering findings in the field of lung cancer.

Treatment strategies for esophageal and EGJ ACs have mainly been developed in Western countries. In the CROSS trial, which was conducted in a cohort comprising 75% of patients with esophageal or EGJ AC, the effect of neoadjuvant chemoradiotherapy tended to be lower for AC than for SCC [25]. A high ratio (28.6%) of hematogenous recurrence after neoadjuvant chemoradiotherapy was also noted [13]. The FLOT4-AIO trial demonstrated a significantly enhanced outcome with perioperative FLOT (docetaxel, oxaliplatin, leucovorin, and 5-FU) therapy for EGJ and gastric AC compared with perioperative ECF/ECX (MAGIC) therapy [26]. For esophageal or EGJ cancer after neoadjuvant CRT, significantly longer disease-free survival for both AC and SCC was associated with adjuvant nivolumab therapy in the Checkmate 577 trial [27]. In the Neo-AEGIS trial, trimodality therapy was directly compared with perioperative chemotherapy for AC of the esophagus and EGJ [28]. This study ended in an equipoise, with significantly fewer liver and lung metastases in the perioperative chemotherapy group.

The current study indicated that EGJ AC may exhibit a greater tendency toward non-lymphogenous progression than EGJ SCC. This may be a favorable factor for treatment emphasizing systemic control since non-lymphogenous progression is more challenging to manage through surgical resection or radiation therapy than progression through the lymphatic system. Taken together, both the findings reported in the literature and the results of the present study suggest that perioperative systemic treatment is promising for patients with EGJ AC.

This study has several limitations, including its retrospective, single-center design. The possibility of selection bias cannot be excluded. The second factor is the variation in treatments. For surgical procedures, the approach and extent of LN dissection, for which a consensus has not been reached, varied between the subgroups, while R0 was secured in all patients. Regarding this, the influence of these factors was considered to be small since both the distribution of sites of LN failure and the incidence of postoperative complications were comparable. The use of perioperative treatment could also have influenced the results. On this point, the numbers of examined and metastatic LNs, as well as the rate of metastatic LNs per examined LNs, were all comparable in both the AC and SCC groups irrespective of neoadjuvant treatment. Therefore, the effect of neoadjuvant treatment to reduce the incidence of LN failure was considered to be limited in the patients who were selected by clinicians’ decision. In addition, neoadjuvant and adjuvant treatments, performed comparably for both groups in total, similarly influenced on incidence of both LN failure and NLR. The third limitation is the relatively small number of patients. Nevertheless, considering the rarity of EGJ SCC and the focus of study which have rarely been investigated before, the value of this study would be certain. Furthermore, this study is the first to note the different patterns and varied timing of postoperative recurrence in EGJ cancer. Future research with larger and more characteristically well-matched cohorts to verify these findings will be desirable and beneficial.

## Conclusions

Compared with EGJ SCC, EGJ AC showed a greater tendency toward non-lymphogenous progression and a greater propensity for longer surgery-to-recurrence periods. Thus, emphasizing perioperative systemic control would be useful for treating resectable locally advanced EGJ AC. In this context, careful attention should be given to the different patterns and timings of recurrence for the different histological subtypes of EGJ cancer in the postoperative follow-up period.

## Data Availability

The research dataset that support the findings of this study is available on reasonable request from the corresponding authors.
